# Some Recent Additions to the Dietary of the Sick

**Published:** 1886-03

**Authors:** R. Shingleton Smith

**Affiliations:** Physician to the Bristol Royal Infirmary


### SOME RECENT ADDITIONS TO THE DIETARY OF THE SICK.

By R. SHINGLETON SMITH, M.D. Lond., F.R.C.P.; Physician to the Bristol Royal Infirmary.

THE great progress in our knowledge of Physiological Chemistry which has been effected during the last quarter of a century should have produced a very decided change in the treatment of disease, and Voltaire's celebrated dictum that the practice of medicine mainly consists in "pouring drugs of which we know little into bodies of which we know less" should be laid on the shelf in company with Albert Smith's medical student and Dickens' Bob Sawyer. "Seeing that the practice of the medical man largely consists in pouring chemicals into that delicately organised vessel, the human body, and that the chemical changes which thereupon take place, or which normally and abnormally occur in it, are certainly not more simple than those which take place in ordinary inert vessels in our laboratories, the necessity for the medical man to have a knowledge of chemistry—and that no slight one—would appear to ordinary minds to stand to reason."*

It is more particularly with regard to digestion that great advances have recently been made—the whole question has been resolved into a problem in chemistry. The composition of the various digestive fluids, their action out of and within the body, their effects on the special components of the food, the preparation of and destructive decomposition of the peptones, and the establishment of the enormous influence which pancreatic secretion exerts in the digestive process,—all these facts have laid the foundation for a scientific treatment of various diseases in which imperfect or difficult digestion plays a conspicuous part. Empiricism can only be abolished by a *complete* knowledge of the chemical processes of the body in all their complex and ever-varying details, and this we cannot claim to have attained; but many of the chemical questions which constantly crop up in dealing with patients can be answered, and many of

* Sir William Armstrong, Brit. Assoc., 1885.

the chemical aberrations corrected or averted, by the scientific method.

No recent improvement has effected so great a transformation in the treatment of disease as the use of *peptonised milk.* It may safely be said that the whole programme of treatment of stomach diseases laid down in our text books is now quite obsolete, and all need to be rewritten. The profession generally has quite accepted and endorsed the principle that the chemical metamorphosis of an insoluble casein, needing gastric digestion, to the soluble peptone, ready for immediate absorption, should be adopted in all cases where gastric digestion is difficult or dangerous in consequence of disease in the stomach itself or elsewhere. Numerous other preparations have been devised for the sake of aiding the powers of digestion in its various parts; among these no preparation takes a higher rank than

CARNRICK'S BEEF PEPTONOIDS. The published analyses show that this is by far the most nutritive of the various meat extracts in common use. It is a fine dry powder, made of meat, wheat gluten, and vaporized milk, containing 87 per cent. of organic substances, and only 7 per cent. of water. Stutzer's analysis has shown that it is remarkable for its high percentage of easily digested albumen, amounting to 56 per cent.; and a not inconsiderable quantity of heat producing elements,—viz., of fat 10·67 per cent., and of soluble non-nitrogenous bodies, such as dextrine and sugar, 10·02 per cent.

On submitting the powder to chemical examination we have found, that it gives a decided reaction in testing for peptones, that it contains phosphates in abundance, and traces of sugar, but no starch.

Its nutritive value, in accordance with its chemical composition, is of a very high standard, and experience shows that its utility is in accord with its chemical characteristics. We have frequently given it to patients, who usually find it to be an efficient nutritive tonic, not unpalatable, and not causing any unpleasant symptoms. It appears to be of great value to those whose digestion is weak, and useful in acute diseases, in the debility of convalescence, as well as in chronic diseases of an organic character.

The superiority of these peptonoids over all other preparations, when the nutritive rather than the stimulating properties of beef extracts are required, is at once obvious on a comparison of the chemical composition of the respective substances; and they should take a high rank in the dietary of the sick, more particularly for those numerous patients who are unable or unwilling to take milk in any form, either plain or peptonised.

CARNRICK'S SOLUBLE FOOD is another useful preparation, which may be given instead of milk to those who are unable to digest the latter. Its analysis shows that it has a high nutritive value, being made up of 50 per cent. of the solid constituents of milk (the casein being brought to a soluble condition by means of pancreatine) and 50 per cent. of wheat (the starch being converted into the soluble form of dextrine and maltose). It has therefore the characteristics of a perfect food, containing all the essential constituents of a normal diet; it is the nearest approach to mother's milk which has been devised; it is already partially digested by means of pancreatine, and is therefore not likely to undergo much coagulation in the stomach: it requires no elaborate preparation, but has only to be boiled for one minute; and it exists in the form of a dry powder, which is not liable to decomposition, and may be always at hand ready for use.

Chemical examination shows that it contains proteids, sugar, and starch; its reaction is neutral. It has no irritating properties, and its taste is of a neutral kind.

We have found it of especial value to persons who have predisposition to flatulence, and in cases of dysphagia its bland and unirritating characteristics cause it to be much appreciated. One patient, who had much difficulty in swallowing any fluids in consequence of deep and extensive tuberculous ulceration of the epiglottis, found that this food suited him better than anything else, and he continued it when all other nourishment was given by enema. Of the numerous varieties of infants' and invalids' foods in the market this appears to be one of the best, and would alone suffice to maintain life, inasmuch as it combines all the essential constituents of food in the proportions necessary for perfect nutrition.

Carnrick's Peptonised Cod Liver Oil and Milk is a preparation which aims at the introduction of cod liver oil in a predigested form easily capable of assimilation. The great value of cod liver oil in the treatment of phthisis and other conditions of defective nutrition is becoming daily more apparent, and is quite in harmony with the fact that fat is an absolute necessity to protoplasmic life. Pure oil can, however, be digested to only a very limited extent: if given in excess, it passes away in semi-saponified masses, or in the oily state. Even in small quantities it often impairs digestion, defeating the very object it seeks to accomplish, and it generally happens that those patients who most need it have the least power to digest it. In many cases the presence and degree of progress is determined and regulated by the ability to take and digest the oily food supplied; if therefore any means can be found for aiding the digestion of the oil, the patient is likely to benefit thereby. Of the many preparations aiming at this object, none appears to attain it so nearly as the combination of cod liver oil with peptonised milk, ingeniously combined by the Maltine Manufacturing Company. The combination forms a very perfect and permanent emulsion, the globules being about 25 per cent. finer than in milk, and of nearly uniform diameter, measuring less than $\frac{1}{4000}$th of an inch. Chemical tests show that it contains much proteid matter, not completely transformed into peptones, but a very slight digestion with liquor pancreaticus completes the transformation. The mixture contains 52 per cent. of the best cod liver oil combined with condensed milk, artificially digested by means of pancreatine, not unpalatable (the taste and smell of the oil being much disguised), and easily assimilable. It should be an invaluable combination in cases where oil is a necessity and the saccharine element no disadvantage, but for diabetic subjects the quantity of sugar present must prohibit its use. Its promoters claim for it that it is an "ideal production," and as an emulsion it must be acknowledged that the claim is well-grounded. But it is more than an emulsion of oil, and accordingly patients may be expected to derive more benefit than from the plain oil, either alone or in combination with artificial digestives: the presence of the proteid and fatty elements of the milk, together with the saccharine matter, increase its nutritive value, and at the same time disguise the taste and aid the digestion of the oil. The experience of those who have employed this compound to any extent in daily practice quite supports the claims of its promoters.

One other preparation—MALTINE—deserves a reference, but it is so well known that it scarcely needs description or comment. The value of malt extract as food in cases of deficient assimilation has been abundantly proved; not only is it in itself nutritious, containing albuminoids and phosphates, but the large proportion of diastase present gives it a digestive value on the starchy elements of other foods. It contains the soluble nitrogenous and nutritive elements of the grain, and is constantly, quickly, and easily assimilated: its nutritive elements are by no means insignificant in value, whilst its diastatic power is a material aid to the digestion of starchy foods. Maltine accordingly is a digestant, and at the same time a food, aiding in digestion and itself contributing to the nourishment of the tissues.

The Maltine Manufacturing Company has done good service to the medical profession and to suffering humanity by the introduction of these various preparations as aids to the debilitated or diseased assimilative organs of the body; they have been well advised in so arranging their combinations that the various elements of the food are all supplied, the peptones more particularly in the Beef Peptonoids, the fatty elements in the Cod Liver Oil and Milk Emulsion, and the saccharine and starchy elements in the Soluble Food and the Maltine. They all appear to effect the purposes for which they have been devised, and we can strongly commend them to the notice of those who are not yet in the habit of prescribing them in the various forms of imperfect digestion and defective assimilation.

